# Coprophagy in Healthy Dogs Does Not Alter Fecal Core Microbiota or Increase Carriage of Enteropathogens

**DOI:** 10.3390/ani16172715

**Published:** 2026-09-01

**Authors:** Rachel Pilla, Maria Chiara Sabetti, Flaminia Antonelli, Matilda Rachele Dametti, Filippo Tagliasacchi, Simona Cannas, Eleonora Fusi, Clara Palestrini, Chiara Locatelli, Jan Suchodolski, Paola Scarpa

**Affiliations:** 1 Department of Veterinary Medicine and Animal Sciences, University of Milan, 26900 Lodi, Italy; flaminia.antonelli@studenti.unimi.it (F.A.); matilda.dametti@unimi.it (M.R.D.); filippo.tagliasacchi@unimi.it (F.T.); simona.cannas@unimi.it (S.C.); eleonora.fusi@unimi.it (E.F.); clara.palestrini@unimi.it (C.P.); chiara.locatelli@unimi.it (C.L.); paola.scarpa@unimi.it (P.S.); 2 Gastrointestinal Laboratory, Department of Small Animal Clinical Sciences, Texas A&M University, College Station, TX 77843, USA; jsuchodolski@cvm.tamu.edu; 3 Department of Veterinary Sciences, University of Parma, 43121 Parma, Italy; mariachiara.sabetti@unipr.it

**Keywords:** coprophagy, microbiota, enteropathogen, canine, dysbiosis index

## Abstract

Coprophagy is a common behavior in domestic dogs, yet its effects on the intestinal microbiota and asymptomatic carriage of enteropathogens remain unclear. This study aimed to determine whether healthy coprophagic dogs differ from non-coprophagic dogs in fecal microbiota composition or prevalence of asymptomatic enteropathogen carriage. Forty-four clinically healthy dogs were prospectively enrolled, including 23 coprophagic and 21 non-coprophagic animals as reported by their owners. Ages spanned from 6 to 119 months old, including 19 mixed-breed dogs and 25 purebred dogs, and diet was not controlled for. Fecal samples were analyzed using quantitative PCR to determine the canine dysbiosis index, quantify core bacterial taxa, and detect selected enteropathogens, including *Clostridium perfringens*, *Clostridioides difficile*, *Campylobacter jejuni*, *Salmonella* spp., canine parvovirus, and toxin-associated genes. Demographic and microbiological data were compared between groups, with correction for multiple testing. No significant differences were observed between coprophagic and non-coprophagic dogs in dysbiosis index (*q* = 1), abundance of the core bacterial taxa evaluated (*q* = 0.17–1), or detection of enteropathogens (*q* = 0.734–1) after adjustment for multiple comparisons. Enteropathogens were absent or detected infrequently in both groups. These findings indicate that coprophagy in otherwise healthy dogs is not associated with measurable alterations in dysbiosis index, core fecal microbiota, or increased enteropathogen carriage.

## Simple Summary

Coprophagy, the consumption of feces, is a common behavior in domestic dogs, but its effects on intestinal health remain poorly understood. Because feces contain large numbers of microorganisms, some people have suggested that coprophagy might change the gut microbiota or increase exposure to harmful intestinal pathogens. We compared healthy dogs that regularly ate feces with healthy dogs that had never been observed performing this behavior. We analyzed their fecal core microbiota using a validated molecular test and screened for several common bacterial and viral enteropathogens. We found no meaningful differences in the intestinal bacterial community or in the detection of enteropathogens between the two groups. These findings suggest that, in otherwise healthy dogs, coprophagy does not appear to alter the gut core microbiota or increase carriage of the pathogens evaluated in this study.

## 1. Introduction

Canine coprophagy, the ingestion of feces, is a common but poorly understood behavior in domestic dogs. Estimated prevalence of coprophagy varies between studies, from about one quarter (23% [1], 26% [2]) to almost half of dogs (44% [3]) being described by their owners as coprophagic. There are two types of coprophagy: autocoprophagy, the act of ingesting self-produced feces, and allocoprophagy, the act of consuming the feces of other animals (including but not limited to other dogs, cats, wildlife, and humans), both of which have been observed in dogs. Consumption of human feces was identified in 56% canine scats in a study of free-ranging dogs in Zimbabwe, suggesting that coprophagy is a significant source of food for domestic canines [4]. This behavior, however, is considered undesirable by most owners, and a common source of complaint due to concerns with parasitism, potential dietary deficiencies, and gastrointestinal health.

The origins and consequences of coprophagy are complex and multifactorial. Several factors have been associated with the development of coprophagic behavior in dogs. These include the presence of other coprophagic animals in the household [5], stress (including prolonged confinement) [6], punishment for feces-eating, feeding frequency (such as only one meal per day), and potentially unbalanced diets, though scientific evidence for dietary causes remains limited [1,7]. Coprophagy may also be more common in multi-dog households and among dogs described as “greedy eaters” [1]. Some hypotheses suggest an evolutionary basis, proposing that the behavior may be inherited from ancestral wolves as a strategy to reduce parasite risk in den areas by consuming fresh feces before parasite eggs become infective [1]. Medical conditions such as exocrine pancreatic insufficiency have also been cited as predisposing factors, though most studies focus on otherwise healthy dogs [7].

The impact of coprophagy on canine intestinal health and microbiota is not fully elucidated. Recent research indicates that coprophagic behavior does not significantly alter fecal pH, short-chain fatty acids, lactic acid, or most measures of nutrient digestibility in healthy adult dogs, suggesting minimal direct impact on gut fermentation products or overall digestive health in the absence of underlying disease [7]. However, coprophagy can complicate the diagnosis of intestinal parasitism, as ingested helminth eggs may pass through the gut and result in false-positive fecal tests [3]. The presence of atypical cysts or eggs, also called spurious parasites, has also been reported in dogs due to ingestion of feces from other species [8,9].

In rabbits and rodents, coprophagy is essential for maintaining a diverse and functionally balanced gut microbiota; restriction of coprophagy leads to decreased diversity and altered microbial composition [8,10,11]. Mouse studies show that coprophagy increases microbial load and diversity in the upper gastrointestinal tract [11]. Fecal microbiota transplantation (FMT), the iatrogenic administration of feces from a healthy animal, has shown similar results in dogs affected by chronic enteropathies, with improved clinical signs and fecal scores, as well as decreased markers of dysbiosis [12,13]. However, there are no direct studies evaluating the impact of spontaneous coprophagy in dogs, and evidence from other species may not directly translate to dogs due to different gastrointestinal tract anatomy and physiology.

While direct studies on the effect of coprophagy on the canine gut microbiota are limited, broader research on the canine gut ecosystem highlights that while disease is the main driver of microbial composition, diet, age, and environment are also relevant, particularly in healthy dogs [14,15,16,17,18]. In other species, coprophagy is known to facilitate the acquisition and maintenance of a diverse and balanced gut microbiota, which is crucial for nutrient absorption, immune function, and protection against pathogens [10,19].

The impact of coprophagy in healthy dogs could also have potential implications for fecal donation in the context of FMT. Current FMT guidelines, including recommendations on criteria for donor selection, do not specifically mention coprophagy [20], possibly due to the paucity of research in the topic. Coprophagy could potentially have beneficial effects due to increased microbial diversity, but it could also carry risks of increased enteropathogen transmission, especially in dogs consuming feces from other dogs.

The effect of coprophagy on the canine microbiome in adult healthy dogs therefore remains an area for further investigation. Therefore, the aim of this study was to compare the abundance of key bacterial species and enteropathogens in healthy dogs that are coprophagic to those who are not coprophagic. Our hypothesis was that dogs who display coprophagic behavior chronically will have differences in core microbiota, and increased asymptomatic carriage of enteropathogens.

## 2. Materials and Methods

### 2.1. Animals

Clinically healthy dogs were enrolled through word of mouth at the University of Milan and University of Parma during routine preventive healthcare visits, including annual vaccination appointments and seasonal prophylactic evaluations. As part of the routine health assessment, all dogs underwent a minimum diagnostic work-up, including a complete physical examination, complete blood count, and serum biochemistry profile.

During clinical examination, owners were asked about gastrointestinal health, and Canine Chronic Enteropathy Clinical Activity Index (CCECAI) scores were calculated. Owners were asked to compile questionnaires including demographics and clinical history. Written informed consent was obtained from all owners, who agreed to the use of left-over samples for research purposes.

Eligible dogs were older than 6 months, had no history of chronic gastrointestinal signs, no antibiotic or probiotic administration in the past three months, and unremarkable physical examination and bloodwork. Exclusion criteria included the presence of clinically relevant systemic or organic diseases or severe abnormalities in clinical examination or laboratory parameters, or uncertain presence or absence of coprophagic behavior based on information provided by the owner. Dogs with isolated mild gastrointestinal abnormalities that were considered physiologically compatible with age or not suggestive of chronic gastrointestinal disease, based on clinical judgment and follow-up, were not excluded.

Following confirmation of eligibility, owners were asked to collect a freely-passed fresh fecal sample within seven days of the clinical evaluation and laboratory testing. Samples were kept at 4 °C and transported with ice packs to the universities, where they were immediately aliquoted and frozen at −20 °C. Because only freely-passed fecal samples were collected for research purposes, no authorization from the Animal Welfare Body was required.

Dogs were classified as coprophagic if owners reported witnessing repeated ingestion of their own feces, or feces from other dogs, cats, humans, and/or wildlife. Dogs were classified as non-coprophagic only when owners reported never having observed ingestion or attempted ingestion of fecal material of any kind during the dog’s lifetime.

A total of 44 healthy dogs were enrolled, with normal clinical examination and clinically unremarkable laboratory parameters (Table 1). Of those, 21 were reported to have never exhibited coprophagic behavior, while 23 were classified as coprophagic. The non-coprophagic group (NCG) included 9 males and 12 females, and the coprophagic group (CG) was comprised of 6 males and 17 females. Ages varied from 8 to 106 months old (median 41 months old) for the NCG, and from 6 to 119 months old (median 38 months old) for the CG. The NCG comprised 10 mixed-breed dogs and 11 purebred dogs (3 Jack Russel Terriers, 3 German Shorthaired Pointers, 2 Australian Shepherds, 1 Nova Scotia Duck Tolling Retriever, 1 Staffordshire Bull Terrier, and 1 Lupino del Gigante). The CG comprised 9 mixed-breed dogs and 14 purebred dogs (4 Labrador Retrievers, 2 Golden Retrievers, and one of each of: English Cocker Spaniel, Boxer, Czechoslovakian Wolfdog, Lagotto Romagnolo, Standard Poodle, Border Collie, Weimaraner, and Chihuahua).

### 2.2. Fecal Analysis

Fecal samples were kept at −20 °C and shipped in dry ice to the Gastrointestinal Laboratory (Texas A&M University, College Station, TX, USA) for analysis. Fecal dysbiosis index (DI) was calculated using qPCR for core bacterial taxa (*Faecalibacterium* spp., *Blautia* spp., *Turicibacter* spp., *Streptococcus* spp., *E. coli*, *Fusobacterium* spp., and *Peptacetobacter* (*Clostridium*) *hiranonis*) as described elsewhere [21]. Other core taxa measured by qPCR included *Bifidobacterium* spp., *Bacteroides* spp., *Prevotella copri*, *Collinsella* spp., and *Ruminococcus gnavus*, as previously described [22], as well as *Akkermansia* spp., *Enterococcus* spp. [23], and the Oscillospiraceae family [24]. *Akkermansia* spp. primers were Akk2F GGGACGACGTCAGGTCAGTA and Akk2R CGTAGCTGATGCGCCATTAC, and PCR conditions were initial denaturation at 95 °C for 3 min, followed by 40 cycles of denaturation at 95 °C for 5 s, annealing at 58 °C for 5 s, and extension at 70 °C for 5 s. Potential enteropathogens were also measured by qPCR and included *C. perfringens*, *C. perfringens* enterotoxin gene, *C. perfringens* NetF toxin gene, *Clostridioides difficile*, canine *Parvovirus*, *Campylobacter jejuni*, and *Salmonella* spp. Primers and qPCR conditions were previously described [25,26]. Cycle threshold (ct) values were converted into logDNA using qPCR-specific formulas generated through standard curves.

### 2.3. Statistical Analysis

Statistical analyses were performed using GraphPad Prism version 10.5.0 for macOS (GraphPad Software, Boston, MA, USA). Demographics and clinical parameters were compared between groups using the unpaired *t*-test (age), chi-square test (sex and purebred vs. mix-breed), Fisher’s exact test (breeds according to the Fédération Cynologique Internationale or FCI breed groups) and Mann–Whitney test (CECCAI).

Dysbiosis index results and logDNA values were tested for normal distribution using the Shapiro–Wilk test, which revealed that only a few datasets were normally distributed. Therefore, they were compared between coprophagic and non-coprophagic dogs using the Mann–Whitney test and adjusted for multiple comparison using Benjamini and Hochberg’s False Discovery Rate [27]. Significance was set as *q* < 0.05.

## 3. Results

### 3.1. Animal Population and Demographics

**Figure 1:**
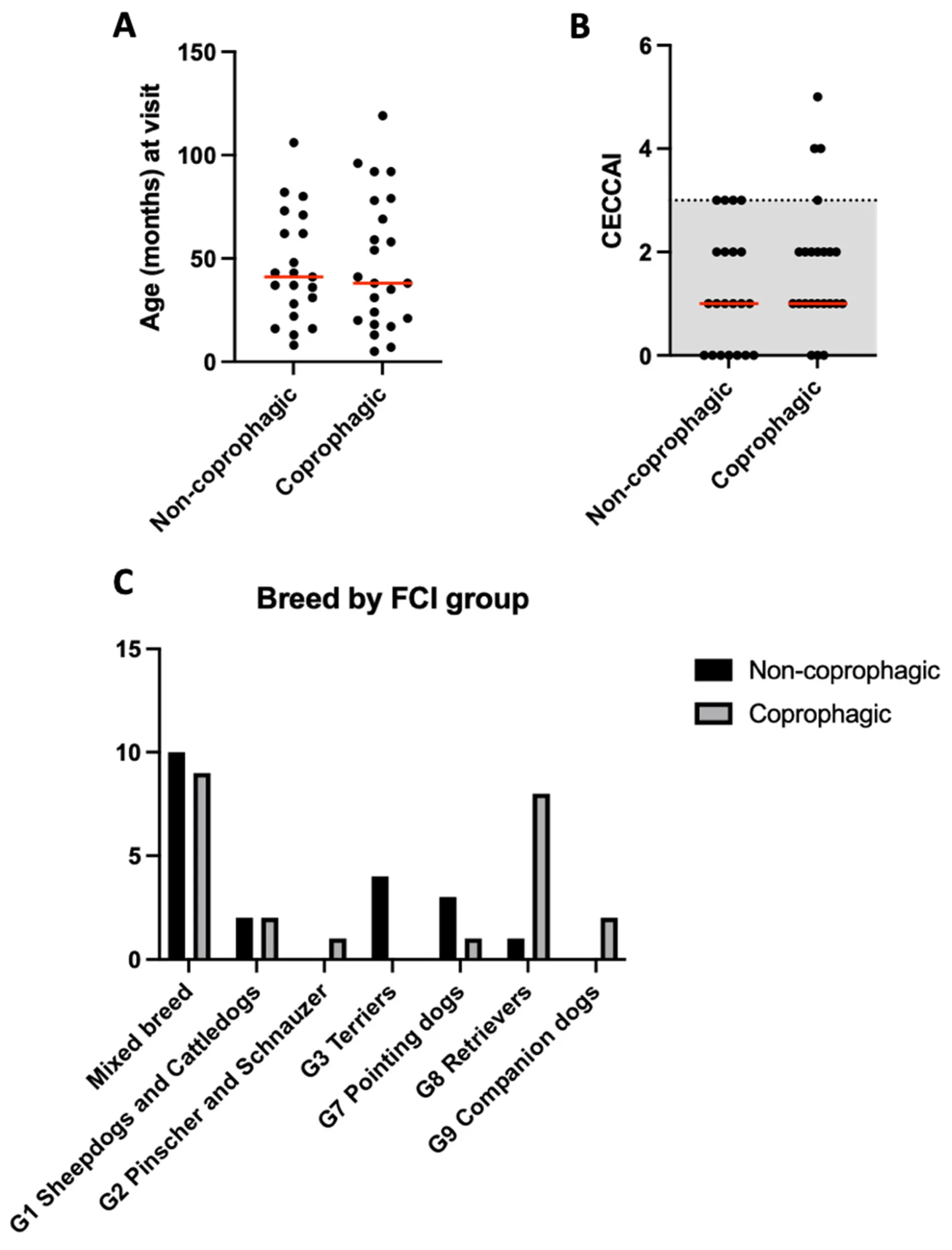
Demographics of enrolled dogs. Age (**A**), CECCAI scores (**B**) and number of dogs per breed groups according to the FCI (**C**) are shown separated by non-coprophagic and coprophagic dogs. Red lines indicate median values.

No differences were observed for sex (*p* = 0.241), age (*p* = 0.779, Figure 1A) or CECCAI scores (*p* = 0.275, Figure 1B) between C and NC dogs. No difference was observed between groups when comparing the frequency of mixed-breed and purebred dogs; however, when purebred dogs were separated by FCI breed groups, statistical significance was observed, with a higher proportion of Groups 3 (Terriers, *n* = 4/4) and 7 (Pointing dogs, *n* = 3/4) in the NCG, and of Groups 8 (Retrievers, *n* = 8/9) and 9 (Companion dogs, *n* = 2/2) in the CG (*p* = 0.016, Figure 1C). For the purpose of this analysis, one dog belonging to the Lupino del Gigante breed, which is not yet recognized by the FCI, was excluded from the analysis.

**Figure 2:**
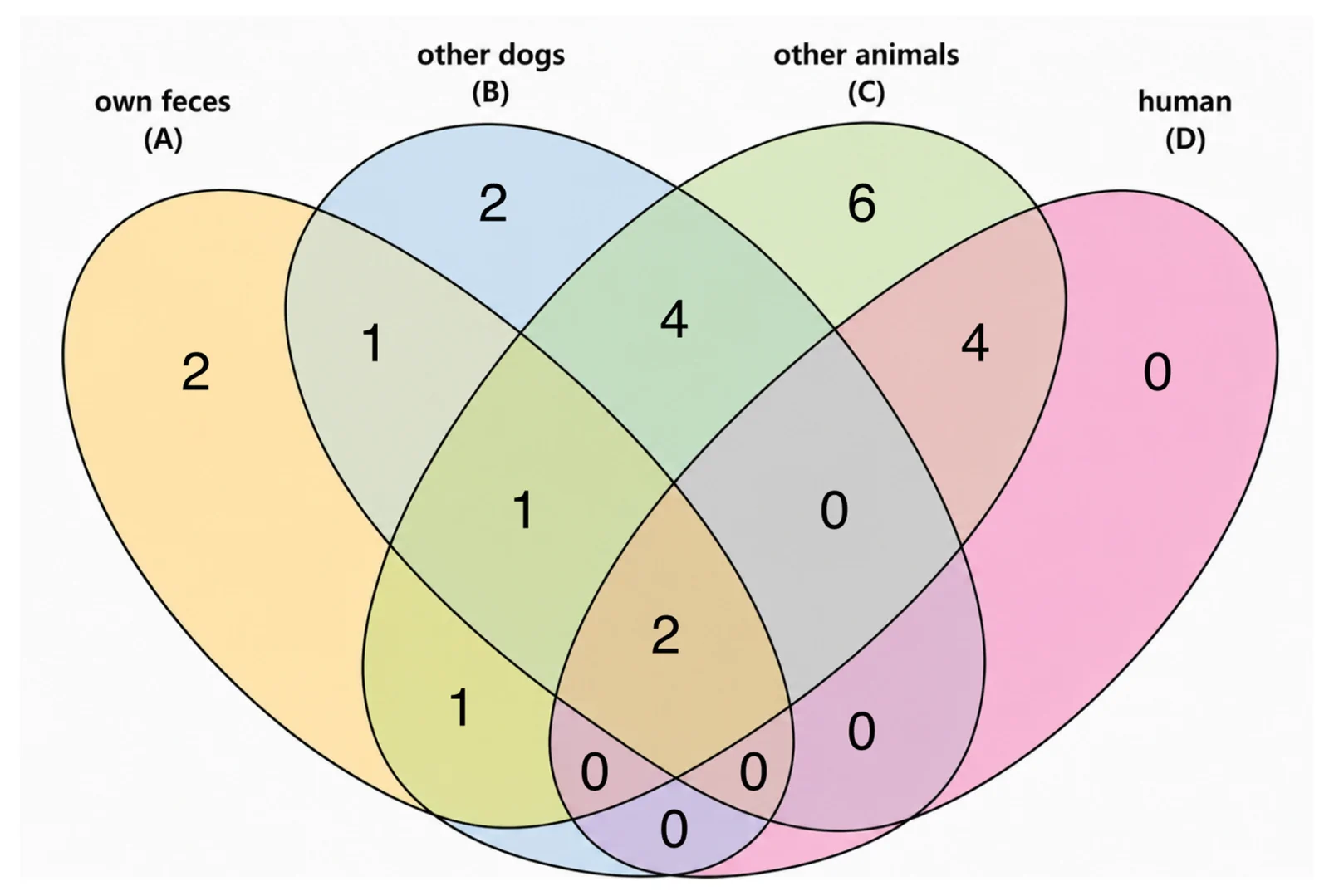
Venn diagram displaying the types of coprophagy observed in the coprophagic group. Autocoprophagy, the ingestion of their own feces, is shown ellipse A; allocoprophagy, the ingestion of conspecific feces, is shown in ellipse B; heterospecific coprophagy, the ingestion of feces from other species, was separated as feces of other animals (ellipse C) and human feces (ellipse D).

The coprophagic dogs were classified based on the type of coprophagy performed (Figure 2). Autocoprophagy, the ingestion of their own feces, was identified in 7/23 dogs (30.4%). Allocoprophagy, the ingestion of conspecific feces, was identified in 10/23 dogs (43.5%). Heterospecific coprophagy, the ingestion of feces from other species, was separated as feces of other animals, identified in 18/23 (78.3%) dogs, and the ingestion of human feces, identified in 6/23 (26.1%) dogs. The most commmon species owners reported seeing their dogs ingesting feces from were cat, nutria, and horses; however, many owners did not specify the animal species or did not know which animal species the feces belonged to. Most dogs (13/23, 56.5%) performed more than one type of coprophagy.

### 3.2. qPCR Results

**Figure 3:**
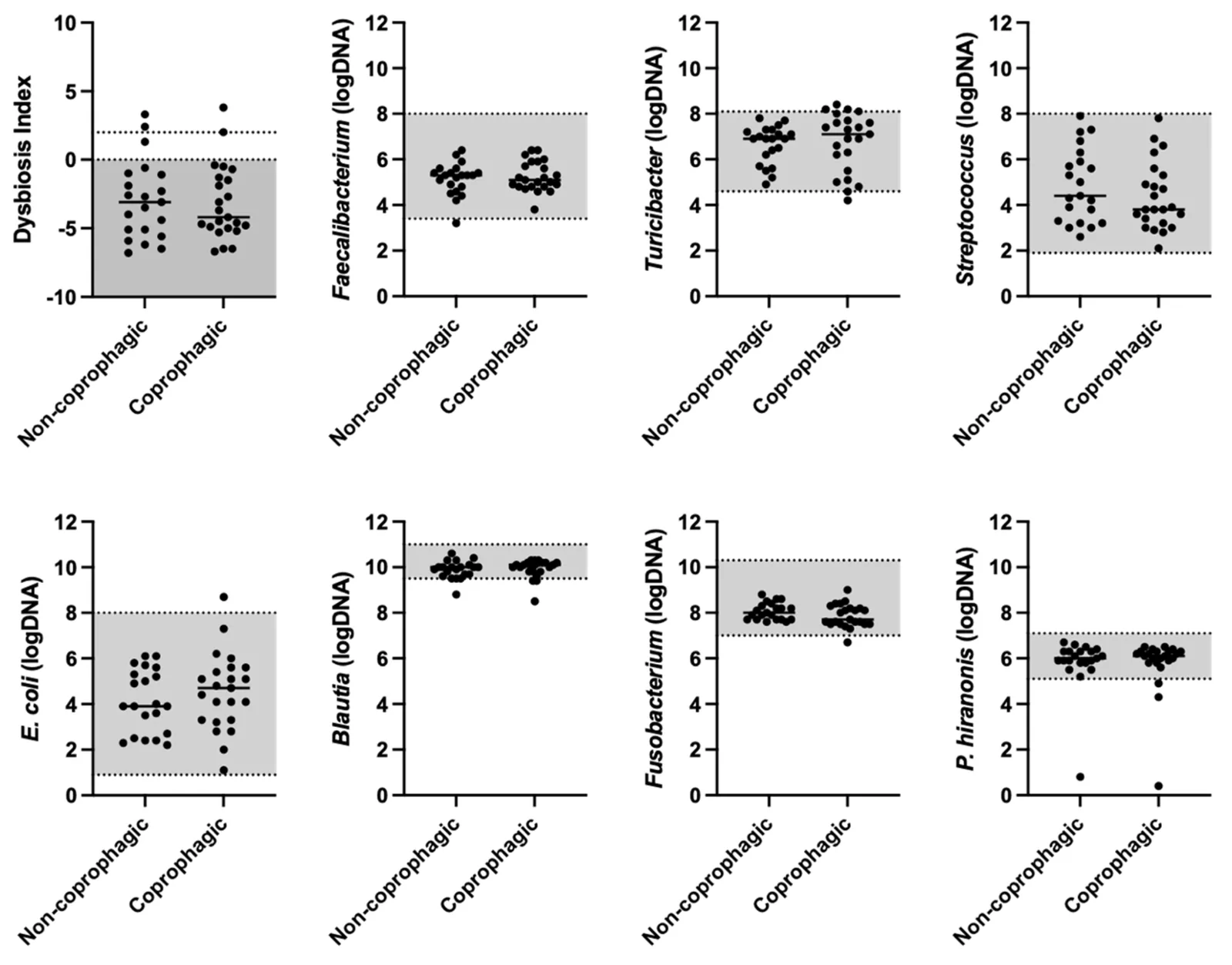
Results of dysbiosis index and the individual taxa included in it. Black lines indicate median values and shaded grey areas indicate reference intervals. No statistical significance was observed between coprophagic and non-coprophagic dogs.

No significant differences were observed between C and NC for DI (*q* = 1), total bacteria (*q* = 0.734), or the core taxa included in the DI. As seen in Figure 3, most healthy dogs fell within reference intervals independently of coprophagy status. *Q*-values for *Faecalibacterium* and *P. hiranonis* were 1, for *Turicibacter*, *Streptococcus*, *E. coli*, and *Blautia* were 0.734, and for *Fusobacterium* was 0.68.

**Figure 4:**
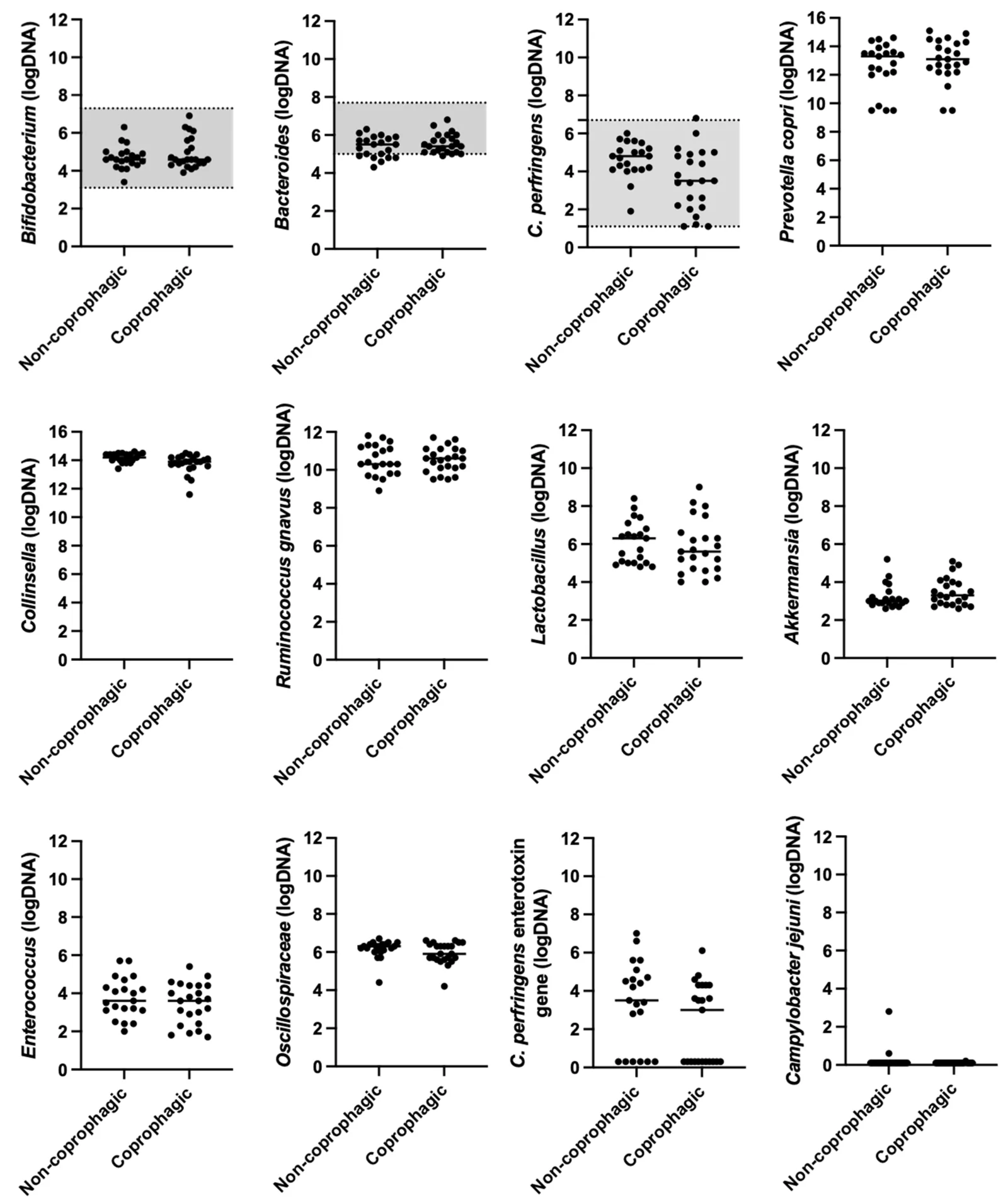
Results of qPCR for core taxa and enteropathogens. Black lines indicate median values and shaded grey areas indicate reference intervals. No statistical significance was observed between coprophagic and non-coprophagic dogs after adjustment for multiple comparisons.

*Clostridium perfringens and Collinsella* spp. had a lower abundance in C dogs (Figure 4); however, significance was lost after adjustment for multiple comparisons (*q* = 0.182 and 0.17, respectively). *C. perfringens* abundance, however, was still within reference intervals for all dogs except for one coprophagic dog. Abundances of *Bifidobacterium*, *Bacteroides*, *Prevotella copri*, *Ruminococcus gnavus*, *Lactobacillus*, *Akkermansia*, *Enterococcus*, and Oscillospiraceae were not different between coprophagic and non-coprophagic dogs (*q* = 0.734 or 1 for all).

*C. perfringens* enterotoxin gene was detected frequently, but no difference was observed between C (12/23, 52.2%) and NC (15/21, 71.4%) dogs (*q* = 0.734). *Clostridioides difficile* and *Campylobacter jejuni* were detected only in 1/21 (4.7%) and 2/21 (9.5%) of NC dogs, and there was no statistical difference (*q* = 0.734 for both). *C. perfringens* NetF gene, canine parvovirus, and *Salmonella* spp. were not detected in any of the samples (*q* = 1 for all).

## 4. Discussion

The goal of this study was to investigate whether clinically healthy dogs exhibiting coprophagic behavior had a different microbiota composition, or a higher risk of asymptomatic enteropathogen carriage. A total of 44 clinically healthy dogs were classified as non-coprophagic (*n* = 21) or coprophagic (*n* = 23), as reported by their owners. Fecal samples were collected and the canine dysbiosis index (DI) was measured, as well as qPCRs for core bacteria and enteropathogens.

No significance was observed in microbiome composition on either the bacteria in the dysbiosis index, or core bacteria measure by qPCR. The dysbiosis index is a clinically validated test to detect dysbiosis, covering key bacterial species responsible for short-chain fatty acid (SCFA) production, including those responsible for butyrate production from both fiber (*Faecalibacterium* spp.) and amino acids (*Fusobacterium* spp.), those responsible for bile acid conversion (*Peptacetobacter hiranonis*), and makers of opportunistic overgrowth (*E. coli* and *Streptococcus* spp.). The DI is indicated in the current FMT guidelines [20] as a recommended screening for FMT donors to exclude animals with abnormal microbiota. Other markers chosen for this study were *Bifidobacterium* spp., *Bacteroides* spp., *Prevotella copri*, *Collinsella* spp., and *Ruminococcus gnavus*, which have been reported as part of the core bacteria of healthy dogs [22], with very few animals under the detection limit using targeted assays. *Akkermansia* spp. was included due to its role as a mucin degrader, which produces metabolites such as short-chain fatty acids and branched-chain fatty acids that are beneficial both for the epithelium and for other commensal microbiota [28]; *Enterococcus* spp. was included as is frequently proposed as a probiotic species, with beneficial effects observed in young puppies [29] but not in adult healthy dogs or dogs with chronic enteropathies [30]; and the Oscillospiraceae family was included due to recent reports of their potential involvement in bile acid conversion [31].

Absolute quantification through qPCR is a reliable, repeatable method that allows for the establishment of reference intervals [21]. Previous studies have shown a strong correlation between the qPCR-based DI and sequencing methods, despite measuring only a subset of the bacteria detected in sequencing [22,32]. That is likely because these bacteria are responsible for key metabolic pathways necessary for a healthy gut environment, which are a pre-requisite for normobiosis. In previous studies in healthy dogs in which differences in microbiome composition due to diet were investigated using both sequencing methods and DI, DI results mirrored those of sequencing, showing that the dysbiosis measured by it is not limited to chronic enteropathies [33,34]. Therefore, while sequencing our samples could have revealed significant differences in taxa not included in our targeted assays, it is unlikely that it would reveal large effect sizes or significant shifts in overall microbiome composition.

Unlike rodents and rabbits, in which coprophagy represents a physiologically relevant behavior [10,11], coprophagy in dogs is generally considered an opportunistic behavioral phenomenon rather than a physiological requirement [4,5,6]. This difference may help explain the absence of measurable alterations in the targeted fecal microbiota observed in the present study.

Enteropathogens were were either not detected or detected infrequently in both groups. The relatively high prevalence of the *Clostridium perfringens* enterotoxin (*cpe*) gene in both groups is consistent with previous studies demonstrating that its carriage may occur in clinically healthy dogs and, by itself, should not be interpreted as evidence of disease [35]. Interestingly, most coprophagic dogs consumed feces from multiple animal species, yet this was not associated with an increased detection of the enteropathogens evaluated in this study. This finding suggests that, in otherwise healthy dogs, occasional exposure to fecal material from other species does not necessarily translate into an increased asymptomatic carriage of the bacterial enteropathogens investigated.

Current FMT guidelines indicate testing for asymptomatic carriage of enteropathogens including *Salmonella* spp. and *C. jejuni*. In addition to those, we chose to test for other potential enteropathogens. *C. difficile*, a dysbiosis-associated pathogen for humans, has a more uncertain role as an enteropathogen in dogs [36]. *C. perfringens* and its toxins, as well as canine parvovirus, were included due to their role in acute diarrhea. *C. perfringens* abundance is often transiently increased in acute diarrhea, and the genes for both its enterotoxin and its NetF toxin have been found to increase in abundance in the early stages of acute hemorrhagic diarrhea syndrome [37]. Canine parvovirus infections are more common in young unvaccinated puppies; however, asymptomatic infections in adult vaccinated dogs is possible [38,39] and can pose a risk when FMT is being used in young diarrhea patients.

A few of the pathogens investigated pose a zoonotic risk, especially to owners who handle and dispose of their dog’s feces on a regular basis. There have been reports of *Salmonella* infections associated with dog ownership, although outbreaks are more frequently associated with handling contaminated pet foods [40] rather than fecal materials. Still, dogs can asymptomatically shed *Salmonella*, a finding more common if they consume raw meats and natural treats; however, no cases of salmonellosis in humans have been reported associated with such practices [41]. In our study we did not observe asymptomatic *Salmonella* shedding in any of the enrolled dogs, suggesting that coprophagy is not a significant predisposing factor for *Salmonella* shedding.

*Clostridioides difficile* strains causing community-acquired infections in humans have been isolated from dogs, leading to a hypothetical concern about zoonotic transmission [42]. Interestingly, *C. difficile*, as in humans, is also associated with dysbiosis and bile acid dysmetabolism in dogs [36]. However, while canine intestinal epithelial cells have been shown to be susceptible to its toxin [43], the toxin is rarely detected in feces of dogs positive for *C. difficile* [36]. The abundance of *C. difficile* has not been correlated with increased disease severity in neither acute nor chronic cases of diarrhea, and response to non-antimicrobial treatment is equivalent between patients who test positive or negative to *C. difficile*, suggesting that no specific treatment is required. Initial studies with 16S rRNA gene sequencing have identified *P. hiranonis* as a protective factor for *C. difficile* infections in dogs [43]. *P. hiranonis* has been identified as a keystone species in the healthy gut microbiome of dogs due to its role as the sole bile acid converting species [44]. Recent studies have highlighted that *C. difficile* positivity in dogs should be considered as a marker of dysbiosis and bile acid dysmetabolism rather than a true infection [36]. In agreement, we found that the only dog positive for *C. difficile* in our study had very low abundance of *P. hiranonis* and a dysbiosis index of 3.3, suggesting the presence of both bile acid dysmetabolism and dysbiosis. Interestingly, that dog was non-coprophagic, suggesting that coprophagy did not increase the risk of *C. difficile* carriage.

*Campylobacter jejuni* was also only identified in two dogs from the NC group. *C. jejuni* is a significant zoonotic concern, with reports of dog owners being infected with the same strain as their dogs [45,46]. Interestingly, *C. jejuni* carriage is more common in puppies rather than adult dogs [45,47], a population not targeted in our study. Puppies are known to have dysbiotic microbiomes in early puppyhood, with microbiome maturation initiating around weaning and approaching the microbiome composition of adult dogs by 10–16 weeks old [25]. In our study we only included 3 puppies, ages 6, 7, and 8 months old, none of which were positive for *C. jejuni*.

Interestingly, we observed a significant difference in breed distribution between groups, with Retrievers (FCI Group 8) and Companion dogs (FCI Group 9) being overrepresented among coprophagic dogs. This finding is consistent with previous reports describing a higher prevalence of coprophagy in dogs characterized as “greedy eaters” [1], a description that fits well with most Labrador and Golden Retrievers. However, care should be taken not to overinterpret this finding, as small numbers of patients can easily skew results. Large-scale studies would be needed to accurately assess breed differences in coprophagic behavior. Importantly, while studies have shown an effect of genetic background and breed in the canine gut microbiome, size effects are generally small and easily overshadowed by medication use, health status, and diet [15,16,17]. Therefore, the demographic asymmetry between groups is unlikely to be a major confounder of our primary microbiological conclusions.

Our study has some limitations, including lack of parasite testing and use of owner-reported data. Unfortunately, parasite testing was not performed at the time of collection, and cannot be performed retrospectively. While the vast majority of the enrolled dogs (41/44, 93.2%) received routine preventative antiparasitic treatments due to living in a heartworm-endemic region, routine prophylaxis may not cover all gastrointestinal protozoa, such as *Giardia* spp., or specific helminths. Given that coprophagy is known to complicate parasite diagnostics and introduce spurious parasites [3,8,9], future studies should include systematic fecal flotation and antigen screening to fully rule out concurrent asymptomatic parasitic infections.

Because our study population comprised pet dogs living in their home environments, diet and living environment could not be controlled for. While those factors can have a significant impact in microbiome composition, size effects are generally small compared to disease, and most healthy dogs receiving different diets remain within the range of normal [48]. This limitation, combined with the small sample size, could mean that subtle or rare differences between groups could have been missed due to low statistical power.

The use of owner-reported data could not be avoided with regards to coprophagic behavior. While initially we had planned to only enroll non-coprophagic dogs that had no unleashed outdoor access, finding non-coprophagic healthy dogs proved very challenging. During enrollment we were hence forced to rely on owner’s reports of never having witnessed any coprophagy or any attempts to consume fecal material. It is therefore possible that some dogs in the non-coprophagic group are hidden coprophages, by either performing coprophagy only sporadically, or by having learned to hide their behavior from humans. In addition, the frequency and quantity of coprophagic behavior were not objectively quantified, as well as the distance between the last coprophagic event and sampling time. Therefore, dose-dependent and acute effects of ingested fecal material cannot be excluded. Lastly, heterogeneity of the ingested material is an inherent limitation. While it would be interesting to isolate the effects of allocoprophagy versus autocoprophagy, a significant proportion of enrolled dogs performed both, which prevented us from investigating this aspect.

One of the motivations of the study was the concern over the use of coprophagic fecal donors for FMT, due to the lack of information regarding the impact of coprophagy in microbiome composition. Current FMT guidelines [20] do not mention coprophagy, but anecdotally, FMT donors often display coprophagic behavior. In our study, coprophagy alone does not appear to justify exclusion of an otherwise healthy donor candidate, provided that all other recommended donor screening criteria are fulfilled.

## 5. Conclusions

Coprophagic behavior in healthy dogs, regardless of the source of the ingested feces, did not significantly impact core microbiota abundance or enteropathogen carriage. There were no significant differences in age or sex between coprophagic and non-coprophagic dogs; however, we did observe a significant difference in purebred dogs, which, despite small numbers, were more likely to display coprophagic behavior if they belonged to FCI groups 8 (Retrievers) and 9 (Companion dogs).

## Figures and Tables

**Table 1:** Laboratory parameters of enrolled dogs in the coprophagy (C) and non-coprophagy (NC) groups. Statistics were performed with Mann–Whitney test and adjusted for multiple comparisons. ^1^ *n* = 40 due to lack of leftover serum.

Test	Median NC	Min NC	Max NC	Median C	Min C	Max C	*q*-Value
Red blood cells (×10^6^/µL)	6.67	4.98	8.03	6.46	4.81	7.96	0.894
Hemoglobin (g/dL)	16.7	11.7	20.2	15.9	12.5	20.1	0.890
Hematocrit (%)	46.4	34.4	54.9	44.2	34.8	54.5	0.890
MCH (pg)	24.8	21.4	26.5	24.6	23.1	26.5	0.936
MCHC (g/dL)	35.8	33.7	36.8	35.85	33.8	36.9	0.894
MCV (µ^3^)	69.7	65.6	87	69.3	65.6	72.9	0.893
Reticulocytes (×10^3^/µL)	37.5	18	118	27.1	9	80	0.890
Platelets (×10^3^/µL)	240	144	487	243	101	350	0.957
White blood cells (×10^3^/µL)	8.89	4.66	13.7	8.595	4.7	16	0.894
Neutrophils (×10^3^/µL)	5.5	3.28	9.03	5.125	2.28	8.65	0.890
Eosinophils (×10^3^/µL)	0.55	0.12	1.99	0.55	0.1	3.5	0.894
Lymphocytes (×10^3^/µL)	2.15	1.02	5.73	2.525	1.26	4.8	0.890
Monocytes (×10^3^/µL)	0.49	0.2	1.1	0.55	0.1	1.15	0.936
Basophils (×10^3^/µL)	0	0	1.4	0	0	0.1	0.894
Cobalamin (ng/L) ^1^	491.5	150	1342	522	192	2000	0.890
Folate (µg/L) ^1^	8.2	2.6	40.2	7	3.6	18.9	0.936
Urea (mg/dL)	39	25	81	39	15	58	0.894
Creatinine (mg/dL)	1.18	0.49	1.6	1.14	0.6	1.8	0.936
Glucose (mg/dL)	94	63	169	94	62	121	0.890
Total protein (g/dL)	6.2	5.5	7.4	6.35	5.3	7.4	0.890
Albumin (g/dL)	3.3	2.3	3.89	3.255	2.7	3.8	0.893
Albumin:globulin ratio	1.16	0.7	1.6	1.1	0.8	1.39	0.890
Calcium (mg/dL)	9.8	8.4	10.7	10	8.8	11.4	0.890
ALT (U/L)	35.5	16	170	39	27	144	0.890
ALP (U/L)	57	32	214	48	15	183	0.890

## Data Availability

The raw data supporting the conclusions of this article will be made available by the authors on request.
